# Sequential treatment escalation improves survival in patients with Waldenstrom macroglobulinemia

**DOI:** 10.1097/BS9.0000000000000179

**Published:** 2024-01-17

**Authors:** Ying Yu, Wenjie Xiong, Tingyu Wang, Yuting Yan, Rui Lyu, Qi Wang, Wei Liu, Gang An, Weiwei Sui, Yan Xu, Wenyang Huang, Dehui Zou, Jianxiang Wang, Lugui Qiu, Shuhua Yi

**Affiliations:** a State Key Laboratory of Experimental Hematology, National Clinical Research Center for Blood Diseases, Haihe Laboratory of Cell Ecosystem, Institute of Hematology& Blood Diseases Hospital, Chinese Academy of Medical Sciences & Peking Union Medical College, Tianjin, China; b Tianjin Institutes of Health Science, Tianjin, China

**Keywords:** Prognosis, Relapse, Treatment options, Waldenstrom macroglobulinemia

## Abstract

Waldenstrom macroglobulinemia (WM) is a type of incurable, indolent B-cell lymphoma that is prone to relapse. Over time, treatment strategies have progressed from cytotoxic drugs to rituximab (R)- or bortezomib (V)-based regimens, and have now entered into an era of Bruton tyrosine kinase inhibitor (BTKi)-based regimens. However, the optimal treatment for the relapsed patients is still unclear. Herein, we analyzed the outcomes of the first- and second-line therapies in 377 patients with WM to illustrate the optimal choices for second-line therapy. After a median follow-up of 45.4 months, 89 patients received second-line therapy, and 53 patients were evaluated for response. The major response rates (MRR) of first- and second-line treatment were 65.1% and 67.9% (*P* = 0.678). The median progression-free survival (PFS) for the second-line therapy (PFS2) was shorter than that for the first-line therapy (PFS1) (56.3 vs 40.7 months, *P* = 0.03). However, PFS2 in targeted drugs group (R-/V-/BTKi-based regimens) was comparable to PFS1 (60.7 months vs 44.7 months, respectively, *P* = 0.21). Regarding second-line therapy, patients who underwent sequential treatment escalation—such as transitioning from cytotoxic drugs to R-/V-/BTKi-based regimens or from R-/V-based to BTKi-based regimens (escalation group) —had higher MRR (80.6% vs 47.1%, respectively, *P* = 0.023) and longer PFS2 (50.4 vs 23.5 months, respectively, *P <* 0.001) compared to the non-escalation group. Patients in the escalation group also had longer post-relapse overall survival compared with the non-escalation group (median, not reached vs 50.7 months, respectively, *P* = 0.039). Our findings indicate that sequential treatment escalation may improve the survival of patients with WM.

## 1. INTRODUCTION

Waldenstrom macroglobulinemia (WM), a B-cell malignancy, is characterized as an immunoglobulin M-secreting lymphoplasmacytic lymphoma according to the World Health Organization criteria.^1^ WM is relatively rare, especially among Asians, and it accounts for only 1% to 2% of all hematological malignancies.^2^ The incidence of WM is higher among individuals of white ethnicity, with an annual rate of 4.1 cases per million, compared to that in other racial groups, where the annual rate is 1.8 cases per million.^2^

The medical therapy for WM has evolved through three noteworthy stages: the era of cytotoxic drugs, represented by medications such as cyclophosphamide, doxorubicin, vincristine, and prednisolone (CHOP), chlorambucil, and fludarabine; the era of new drug therapy, which includes rituximab (R) and proteasome inhibitors; and the era of targeted drug therapy, featuring medications such as Bruton tyrosine kinase inhibitors (BTKi), B-cell lymphoma-2 (BCL-2) inhibitors, and phosphatidylinositol 3-kinase inhibitors.^3–5^

Currently, an increasing number of treatment options for WM are available, and they have demonstrated relatively high efficacy.^4,6^ However, due to its incurable nature, patients with WM experience a relapse over time. Targeted drugs, such as BTKi and BCL-2 inhibitors, have also demonstrated high efficacy in patients with relapsed WM.^4,7^ According to the latest National Comprehensive Cancer Network guidelines and consensus from the International Workshop for WM, the recommended regimen for second-line therapy is nearly the same as that for first-line therapy.^8,9^ However, the optimal approach to maximizing the survival of patients with WM through sequential treatment escalation remains unknown. Therefore, we conducted a retrospective, single-center study, spanning a period of 20 years, on 377 patients with WM to investigate the treatment options and relapse patterns of WM.

## 2. MATERIALS AND METHODS

### 2.1. Patients

Between June 2000 and September 2021, 447 patients were diagnosed with lymphoplasmacytic lymphoma (LPL)/WM at the Blood Disease Hospital, Chinese Academy of Medical Sciences & Peking Union Medical College. Of these, 396 were newly diagnosed cases. Seven symptomatic individuals who refused any treatment and 12 patients who remained asymptomatic during the course of the study were excluded. Finally, 377 patients with newly diagnosed WM who had received systemic treatment were included in this analysis. Written informed consent was obtained from each patient, and the study was approved by the institutional ethics committee (IIT2021030-EC-1). The study complied with the ethical standards of the 1964 Declaration of Helsinki and its later amendments.

The diagnosis was reviewed and confirmed by 2 hematopathologists according to the consensus panel definition of WM.^10^ The following baseline data, that is, complete clinical and biological data of all patients were available at the time of diagnosis: age; sex; date of diagnosis of WM; time from diagnosis of WM to treatment initiation; baseline laboratory data, such as complete blood count, serum concentrations of β2-microglobulin, albumin, immunoglobulin M, and serum protein electrophoresis; chromosomal study; fluorescence in situ hybridization; and evaluations for lymphadenopathy, splenomegaly, and bone marrow infiltration.

### 2.2. Treatment

All symptomatic patients in our hospital were treated using either cytotoxic drugs, chemotherapy with rituximab (R) and/or bortezomib (V), or targeted drugs (BTKi). We classified the treatment options for all the patients into the following 4 regimens:

(i). R-based regimens: rituximab, cyclophosphamide, and dexamethasone (RCD); rituximab, CHOP (R-CHOP); rituximab, cyclophosphamide, vincristine, and prednisolone (RCOP); rituximab, fludarabine plus cyclophosphamide (RFC), rituximab plus bendamustine (BR); and bortezomib, rituximab, and dexamethasone (BRD)
(ii). V-based regimens: bortezomib and dexamethasone (BD); and bortezomib, cyclophosphamide, and dexamethasone (BCD)
(iii). BTKi-based regimens: BTKi single-agent or combination therapies
(iv). Cytotoxic drugs: chlorambucil, CHOP, COP, fludarabine plus cyclophosphamide (FC), and thalidomide plus cyclophosphamide and dexamethasone (TCD).

The details of the regimens are shown in Table S1, http://links.lww.com/BS/A79. We classified patients who received R-, V-, and BTKi-based regimens into the “targeted drugs group,” and those who received cytotoxic drugs into the “conventional cytotoxic drugs group.” Patients who received at least 2 courses of treatment were considered to have received systematic treatment and could be included in the treatment groups.

### 2.3. Efficacy evaluation and outcomes

Assessment of the treatment response in patients with measurable WM was based on the NCCN guidelines (Version 2.2022) of LPL/WM and the latest consensus from the 11th International Workshop on WM.^8,11^ The overall response rate (ORR) encompassed the minor response (MR), partial response (PR), very good partial response (VGPR), and complete response (CR) rates. Major response rate (MRR) was defined as the sum of CR, VGPR, and PR.

Progression-free survival (PFS) was defined as “the time from treatment to disease progression or death from any cause.” PFS2 was defined as “the time from second-line treatment to second disease progression or death.” Overall survival (OS) was measured as “the interval between the date of diagnosis and the date of death or last follow-up.” Post-relapse OS was measured as “the interval between the date of disease progression and the date of death or last follow-up.”

### 2.4. Statistical analysis

Data on patient characteristics were summarized using median and range for continuous variables and absolute and relative frequencies for categorical variables. Pearson Chi-square test and Fisher exact test were used to assess the association between the 2 categorical variables. The comparison of quantitative variables between the 2 independent groups of patients was evaluated using the independent samples *t* test. Survival curves were calculated using the Kaplan–Meier method, with differences estimated using the log-rank test.

Statistical significance was set at *P* < 0.05. All the statistical analyses were performed using IBM SPSS software (version 26.0; IBM Corporation, Armonk, New York) and/or GraphPad Prism software (version 8.0; GraphPad Software, Inc., La Jolla, California).

## 3. RESULTS

### 3.1. Patient characteristics

The median age of the 377 patients was 62 years (range: 28–87 years) with a male to female ratio of 2.6:1 and a median immunoglobulin M level of 3120 mg/dL. The median hemoglobin level was 8.4 g/dL (range: 2.4–18.7), and 80.1% of the patients were diagnosed with anemia. The proportion of patients in the low-, intermediate-, and high-risk groups of the International Prognostic Scoring System for WM was 19.3%, 39.2%, and 41.5%, respectively. The percentages of the patients with hepatomegaly, splenomegaly, and lymphadenopathy were 19.8%, 47.8%, and 31.4%, respectively. The median percentage of abnormal lymphoplasmacytic cells detected by flow cytometry was 10.4% (range: 0.2%–91.2%). The basic clinical characteristics of the patients are presented in Table 1.

**Table 1:** Clinical characteristics of the 377 patients with lymphoplasmacytic lymphoma/Waldenstrom macroglobulinemia receiving systemic treatment.

Characteristic	Patients (N = 377)
Age (y)	
Median (range)	62 (22–87)
≥65, n (%)	136 (36.1)
Sex, n (%)	
Male	273 (72.4)
Female	104 (27.6)
Waldenstrom macroglobulinemia IPSS score, n (%)	
Low	68/352 (19.3)
Intermediate	138/352 (39.2)
High	146/352 (41.5)
Waldenstrom macroglobulinemia RIPSS score, n (%)	
Very low	46/339 (13.6)
Low	98/339 (28.9)
Intermediate	124/339 (36.6)
High	57/339 (16.8)
Very high	14/339 (4.1)
IgM level	
Median (range), mg/dL	3120 (120–14,400)
≥7000 mg/dL, n (%)	57 (15.1)
Cytopenia at baseline, n (%)	
Hemoglobin of ≤11 g/dL	302 (80.1)
Platelet count of ≤100,000/mm^3^	129 (34.2)
Absolute neutrophil count of ≤1500/mm^3^	54/355 (15.2)
Median hemoglobin (range), g/dL	8.4 (2.4–18.7)
Percentage of bone marrow abnormal cells of FCM, median (range) %	13.4 (0.2–92.9)
Abnormal B cells, median (range) %	10.37 (0.2–91.2)
Abnormal plasma cells, median (range) %	0.37 (0–12.7)
Median β2 microglobulin (range), mg/L	4.12 (1.0–25.6)
>3 mg/L, n (%)	227/307 (73.9)
Median lactic dehydrogenase (range), U/L	147 (52–874)
≥250 U/L, n (%)	51/346 (14.7)
Median serum albumin (range), g/L	34.1 (19.1–49.8)
<35 g/L, n (%)	
Extramedullary disease, n (%)	
Splenomegaly ≥13 cm	165/345 (47.8)
Hepatomegaly	68/343 (19.8)
Lymphadenopathy ≥1.5 cm	76/242 (31.4)

FCM = flow cytometry, IgM = immunoglobulin M, IPSS = international prognostic scoring system for WM, RIPSS = revised international prognostic scoring system for WM, WM = Waldenstrom macroglobulinemia.

The most common symptoms at the time of diagnosis were fatigue (53.8%) and bleeding (9.5%). Hyperviscosity was observed in 21 (5.6%) patients. Other symptoms are shown in Table S2, http://links.lww.com/BS/A79.

### 3.2. Treatment patterns and responses of first-line therapy

**Figure 1.**
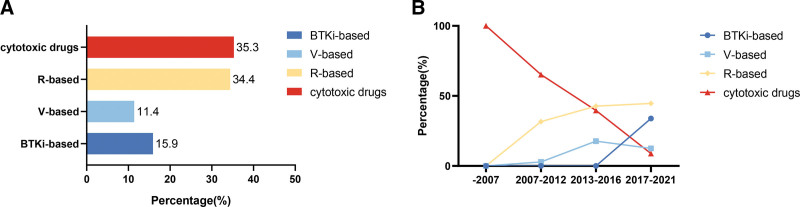
Treatment patterns of first-line therapy. (A) Proportion of patients with Waldenstrom macroglobulinemia treated with each of the 4 regimens. (B) The proportion of patients that received the following different treatment regimens in different eras: cytotoxic drugs, R, V, BTKi. BTKi = Bruton tyrosine kinase inhibitors, R = rituximab, V = bortezomib.

Among the 377 patients who received systemic treatment, cytotoxic drugs (35.3%) and R-based regimens (34.4%) were the most common treatment options. Moreover, V-based and BTKi-based regimens were administered to 11.4% and 15.9% of the patients, respectively (**Fig. 1A**).

Treatment regimens for WM have changed radically over the last 20 years. Before 2007, cytotoxic drugs were the only available option. Between 2007 and 2012, although bortezomib and rituximab were initially administered, cytotoxic drugs continued to be considered the main treatment regimen. Between 2013 and 2016, the number of R- and B-based regimens increased significantly. Since 2017, BTKi has become an important treatment option for WM in China, and more than 90% of the patients have received targeted drugs. Figure 1B shows the prevalence of the various treatment regimens over time.

Among the patients who received systemic treatment, 315 were available for response evaluation. The ORR was 78.7%, which included a CR, VGPR, PR, and MR of 6.7%, 11.7%, 46.7%, and 13.7%, respectively, with an MRR of 65.1%. The ORR and MRR in the targeted drugs group were significantly higher than those in the conventional cytotoxic drugs group (ORR: 84.7% vs 65.7%, *P* < 0.001; MRR: 71.8% vs 50.5%, *P* < 0.001) (Table 2).

**Table 2:** The efficacy of the 4 treatment regimens in first-line therapy.

Regimens	CR (%)	VGPR (%)	PR (%)	MR (%)	SD (%)	MRR (%)	ORR (%)
Cytotoxic drugs (N = 99)	3 (3.0)	5 (5.1)	42 (42.4)	15 (15.2)	34 (34.3)	50 (50.5)	65 (65.7)
R-based (N = 129)	13 (10.1)	22 (17.1)	61 (47.3)	15 (11.6)	18 (14.0)	96 (74.4)	111 (86.0)
V-based (N = 41)	4 (9.8)	3 (7.3)	20 (48.8)	8 (19.5)	6 (14.6)	27 (65.9)	35 (85.4)
BTKi-based (N = 46)	1 (2.2)	7 (15.2)	24 (52.2)	5 (10.9)	9 (19.6)	32 (70.0)	37 (80.4)
Total (N = 315)	21 (6.7)	37 (11.7)	147 (46.7)	43 (13.7)	67 (21.2)	205 (65.1)	248 (78.7)

BTKi = Bruton tyrosine kinase inhibitor, CR = complete remission, MR = minor response, MRR = major response rate, ORR = overall response rate, PR = partial response, R = rituximab, SD = stable disease, V = bortezomib, VGPR = very good partial response.

### 3.3. Survival following first-line therapy

**Figure 2.**
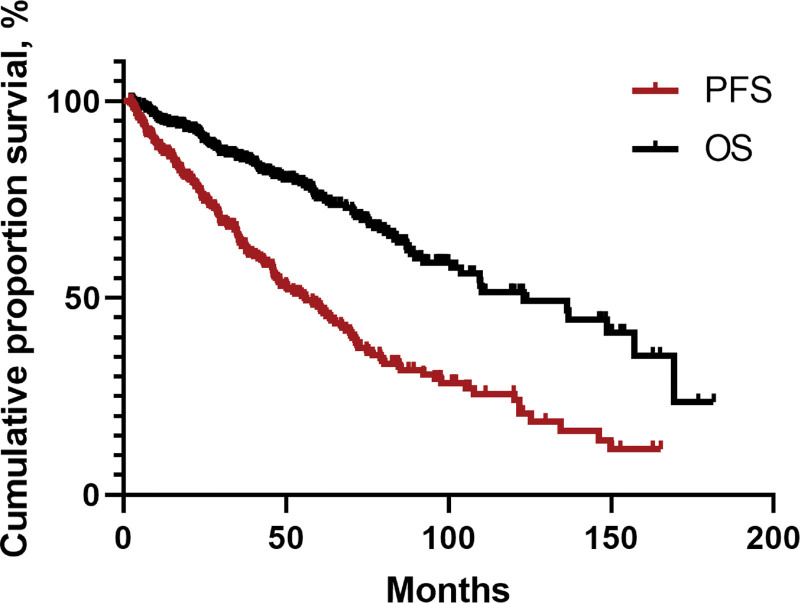
PFS and OS of the 377 patients with Waldenstrom macroglobulinemia. PFS = progression-free survival, OS = overall survival.

**Figure 3.**
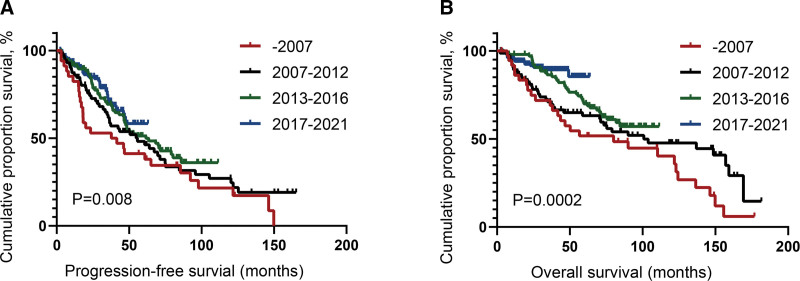
Survival outcomes of patients with WM in different eras. (A) Progression-free survival of patients. (B) Overall survival of patients. WM = Waldenstrom macroglobulinemia.

**Figure 4.**
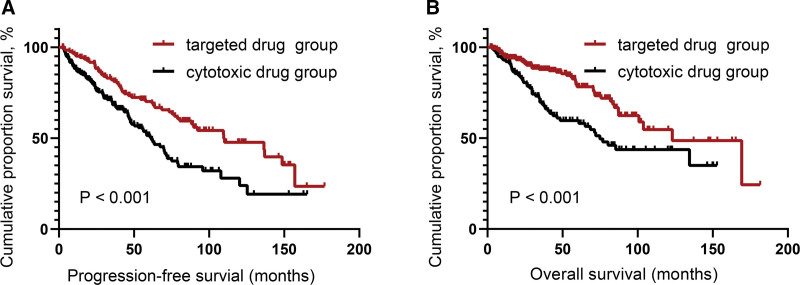
Survival outcomes of patients with WM who received targeted drugs and conventional cytotoxic drugs. (A) Progression-free survival of the patients. (B) Overall survival of the patients. WM = Waldenstrom macroglobulinemia.

At a median follow-up of 45.4 months (95% confidence interval [CI]: 41.1–52.3 months), the median PFS and OS for the 377 patients were 56.3 (95% CI: 45.2–64.3) months and 123.0 (95% CI: 95.7–150.3) months, respectively. The 5-year PFS and OS rates were 47.8% and 75.3%, respectively (**Fig. 2**). Notably, PFS and OS significantly increased with the evolution in the treatment regimens for WM over the eras (**Fig. 3A, B**). Patients in the targeted drugs group exhibited better outcomes than those in the conventional cytotoxic drugs group (median PFS: 109.7 vs 61.6 months, *P* < 0.001; median OS: 123.0 vs 74.8 months, *P* < 0.001) (**Fig. 4A, B**).

### 3.4. Treatment responses and outcomes of second-line therapy

A total of 89 patients received second-line therapy, and 53 of these were evaluated for response. BTKi-based regimens were the predominant treatment option, administered to 47.2% (42/89) of the patients. Cytotoxic drugs and R-based regimens were administered to 22.5% (20/89) of the patients. Seven patients (7.9%) received the V-based regimens. Following second-line therapy, none of the patients achieved CR, 8 (15.1%) achieved VGPR, 28 (52.8%) achieved PR, 8 (15.1%) achieved MR, and 9 (17.0%) exhibited stable disease (SD). The ORR was 83.0%, and the MRR was 67.9% for second-line treatment. Among the 4 treatment regimen groups, the BTKi-based regimens had the highest MRR (80.6%) and ORR (96.8%) (Table 3).

**Table 3:** The efficacy of the 4 treatment regimens in second-line therapy.

Regimens	CR (%)	VGPR (%)	PR (%)	MR (%)	SD (%)	MRR (%)	ORR (%)
Cytotoxic drugs (N = 11)	0	0	4 (36.3)	2 (18.2)	5 (45.4)	4 (36.3)	6 (54.5)
R-based (N = 8)	0	1 (12.5)	5 (62.5)	0	2 (25.0)	6 (75.0)	6 (75.0)
V-based (N = 3)	0	0	1 (33.3)	1 (33.3)	1 (33.3)	1 (33.3)	2 (66.7)
BTKi-based (N = 31)	0	7 (22.6)	18 (58.1)	5 (16.1)	1 (3.2)	25 (80.6)	30 (96.8)
Total (N = 53)	0	8 (15.1)	28 (52.8)	8 (15.1)	9 (17.0)	36 (67.9)	44 (83.0)

BTKi = Bruton tyrosine kinase inhibitor, CR = complete remission, MR = minor response, MRR = major response rate, ORR = overall response rate, PR = partial response, R = rituximab, SD = stable disease, V = bortezomib, VGPR = very good partial response.

### 3.5. Comparison of treatment outcomes between first- and second-line therapy

Compared with first-line therapy, no significant differences were observed in the MRR (67.9% vs 65.1%, *P* = 0.687) and ORR (83.0% vs 78.7%, *P* = 0.475) for second-line therapy. Subgroup analysis of each of the 4 treatment regimens also showed no significant difference in the MRR between first- and second-line therapy (cytotoxic drugs, *P* = 0.37; R-based, *P* = 0.97; V-based, *P* = 0.54; BTKi-based, *P* = 0.31).

The median follow-up duration of the relapsed WM cohort from second-line therapy was 28.6 months (95% CI: 21.6–42.6). PFS2 was significantly decreased compared with PFS1 (median: 56.3 vs 40.7 months, *P* = 0.03). However, the subgroup analysis showed no significant differences in PFS1 and PFS2 among each of the 3 targeted drug regimen groups (median: 60.7 vs 44.7, *P* = 0.21; R-based: *P* = 0.73; V-based: *P* = 0.50; BTKi-based: *P* = 0.45) (Supplementary Figure 1A–C, http://links.lww.com/BS/A79). PFS2 was significantly shorter than PFS1 in the conventional cytotoxic drugs group (Supplementary Figure 1D, http://links.lww.com/BS/A79).

### 3.6. Transition patterns from first- to second-line treatment and their role in response and survival

**Figure 5.**
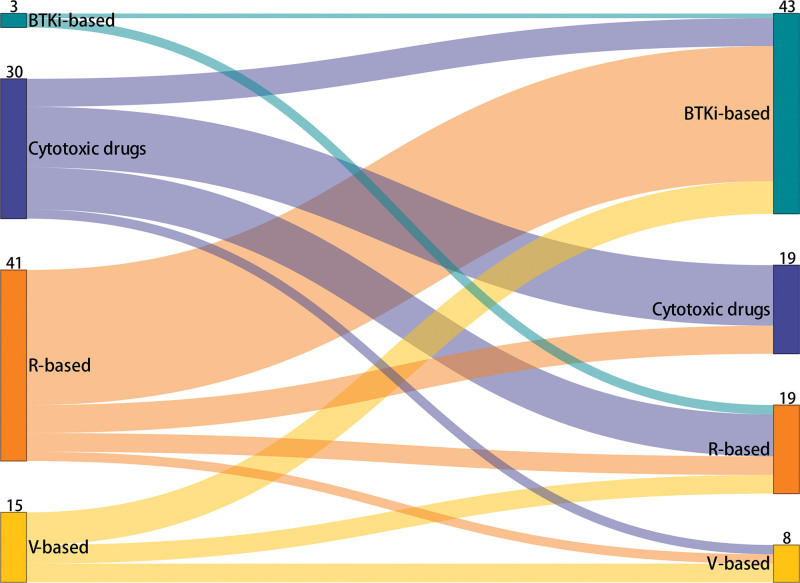
Transition from first- to second-line treatment regimens. BTKi = Bruton tyrosine kinase inhibitors, R = rituximab, V = bortezomib.

We subsequently analyzed the transition patterns from first- to second-line therapy. We ranked the treatment options into 3 stages according to their order of availability: cytotoxic drugs, R- or V-based regimens, and BTKi-based regimens. Patients were classified into the escalation group if the first- and second-line therapy regimens administered to them followed this ranking order, such as transitioning from cytotoxic drugs to R-/V-/BTKi-based regimens, and from R-/V-based to BTKi-based regimens. Patients who did not follow this order were classified into the non-escalation group. Among the 89 patients who received second-line therapy, the most common treatment transition was from R-based to BTKi-based regimens, which was observed in 29 patients (32.6%). Transition from one cytotoxic drug to another was the second most popular treatment pattern (13/89, 14.6%), followed by transition from cytotoxic drugs to R-based regimen (9/89, 10.1%), and from V-based to BTKi-based regimens (7/89, 7.9%) (**Fig. 5**; Table S3, http://links.lww.com/BS/A79).

Among the 53 patients who underwent response evaluation, 36 and 17 patients were from the escalation and non-escalation groups, respectively. The MRR in the escalation group was 80.6% (CR, 0; VGPR, 19.4%; PR, 61.1%), which was significantly higher than the MRR of 47.1% (CR, 0; VGPR, 5.9%; PR, 41.2%) in the non-escalation group (*P* = 0.023).

**Figure 6.**
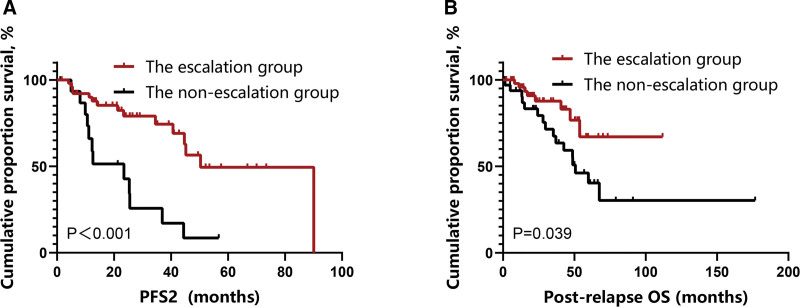
Survival outcomes of patients with WM in the escalation and non-escalation groups. (A) PFS2 of the patients; (B) OS of the patients. OS = overall survival, PFS2 = progression-free survival for the second-line therapy, WM = Waldenstrom macroglobulinemia.

The median follow-up duration following relapse in the escalation group and non-escalation group was 23.3 months (95% CI: 15.2–43.3) and 37.0 (95% CI: 13.4–56.8) months, respectively. The escalation group exhibited longer PFS2 (median, 50.4 vs 23.5 months, *P <*0.001) and post-relapse OS time (median, not reached vs 50.7 months, respectively, *P* = 0.039) than the non-escalation group (**Fig. 6A, B**). Even among the patients who achieved an MRR efficacy with second-line therapy, those in the escalation group also exhibited longer PFS2 than those in the non-escalation group (median: 90.1 vs 23.5 months, *P* = 0.04) (Supplementary Figure 2, http://links.lww.com/BS/A79).

## 4. DISCUSSION

As there is a paucity of research on the best treatment option and prognosis for patients with relapsed WM, in this study, we reported the treatment options, responses, and survival rates of both first- and second-line therapies for patients with WM in China over the last 20 years. Our findings indicate that the MRR and PFS2 were considerably higher in the sequential treatment escalation group than in the non-escalation group for relapsed WM.

Over the past 2 decades, substantial progress has been achieved in the development of novel agents for the treatment of WM. Per the findings of a retrospective study in China, cytotoxic drugs are considered an important treatment option for patients with WM.^12^ Previous studies have indicated that the targeted drugs (R-based, V-based, and BTKi) have high response rates in patients receiving first-line therapy and that the ORR for each treatment reaches over 90%.^5,6,13^ Our data also demonstrated a high remission rate in the targeted drugs group (ORR: 84.7%, MRR: 78.1%), which was significantly higher than that of the conventional cytotoxic drugs group.

For patients who experience a relapse, current therapies encompass a variety of drugs, including immunochemotherapy, proteasome inhibitors, and BTKi.^14^ These first-line regimens also demonstrated high response rates when used as second-line therapies.^7,15,16^ In our study, the subgroup analysis also demonstrated no significant differences in PFS1 and PFS2 among the targeted drug groups. More importantly, the MRR and PFS2 were considerably higher in the escalation group than that in the non-escalation group. These findings suggest that advancements in drug development contribute to the enhancement of survival outcomes in WM.

The latest study from the Mayo Clinic Medical Center similarly focused on the outcomes of patients with relapse and impact of the specific type of therapy used in the second-line setting.^17^ They found that currently available second-line therapies were effective in patients with relapse, as confirmed by similar MRR and ORR among first- and second-line therapies in our study. Moreover, they found that frontline BTKi followed by chemoimmunotherapy as second-line therapy shares similar outcomes to the treatment with initial chemoimmunotherapy followed by BTKi. This finding implies that a treatment change may not always be justified. However, in our study, most of the patients in the non-escalation group were treated with the same or similar regimen as that of the first-line treatment, such as a transition from cytotoxic drugs to cytotoxic drugs. Two patients in our study were transitioned from a BTKi-based to an R-based regimen; one achieved PR efficacy and sustained remission for 21.3 months in second-line therapy; the other patient also achieved PR efficacy again and sustained remission. Details of the outcomes for the patients in the non-escalation group are shown in Table S4, http://links.lww.com/BS/A79. Therefore, our results are not contrary to the aforementioned study, and sequential treatment improved the survival of patients with WM.

Our findings and those of previous studies have demonstrated a median OS of more than 10 years for patients with WM.^14,18^ Moreover, patients with WM continue to demonstrate a high response rate to second-line treatment, and prior lines of therapy or refractory disease have shown to have no significant effect on the response to BTKi.^17,19^ This indicates that treating patients with relapsed WM is not considered challenging. The prolonged survival of patients with WM can be attributed to several factors. One possible reason is the relatively simple genetic changes observed in WM. Unlike multiple myeloma and other lymphomas, the only abnormality of WM thus far identified is chromosome 6q deletion.^20^ In contrast, acute leukemia, multiple myeloma, diffuse large B-cell lymphoma, and other lymphomas are refractory to treatment upon disease progression or relapse. Even with intensive therapy or a combination of targeted drugs, the response rate to treatment is significantly decreased, and the survival time of patients is significantly shortened.^20–22^ Another contributing factor to the long survival of patients with WM is advancements in drug development. The development of several drugs with different mechanisms of action may circumvent the problem of cross-resistance, thereby providing more treatment options for patients. Thus, we should encourage sequential treatment escalation in patients with WM to achieve long-term survival. However, this study has some limitations. First, the retrospective design may have introduced a bias. Second, the number of patients receiving second-line therapy is small and external validation in a larger and independent series of patients receiving sequential treatment is warranted to verify the conclusions.

Overall, the treatment landscape for WM has undergone significant changes with the advent of newer therapies, which has driven survival improvement in patients with WM. However, there is an ongoing need for continued research and exploration of drug therapies to establish a rational treatment strategy and to tailor therapy to meet individual patients’ needs.

## ACKNOWLEDGMENTS

This work was supported by grants from the National Nature Science Foundation of China (81970187, 82170193, 81920108006, and 81900203) and the Chinese Academy of Medical Sciences Innovation Fund for Medical Sciences (2022-I2M-1-022, 2021-I2M-C, and T-B-081).

## AUTHOR CONTRIBUTIONS

S.Y. conceptualized the study design. Y.Y. and W.X. analyzed the data, performed statistical analyses, and wrote the manuscript. T.W., Y.Y., R.L., Q.W., W.L., G.A., W.S., Y.X., W.H., and D.Z. acquired the data and managed the patients. J.W., L.Q., and S.Y. revised the manuscript critically and approved the final version.

## ETHICAL APPROVAL

All procedures performed in studies involving human participants were in accordance with the ethical standards of the institution and with the 1964 Helsinki Declaration and its later amendments or comparable ethical standards.

## Supplementary Material
