# Erratum: Sequential treatment escalation improves survival in patients with Waldenstrom macroglobulinemia

**DOI:** 10.1097/BS9.0000000000000185

**Published:** 2024-03-07

In the original publication of our paper, “Sequential treatment escalation improves survival in patients with Waldenstrom macroglobulinemia,” published on *Blood Science*, 2024 Jan; 6(1): e00179, we have identified several errors that require correction. We apologize for any inconvenience caused and appreciate the opportunity to clarify the following:

In Abstract, the post-relapse overall survival of patients in the escalation group and the non-escalation group (median, not reached vs. 50.7 months, respectively, *P*=0.039) was incorrectly reported.

In Results of 3.6, the post-relapse overall survival of patients in the escalation group and the non-escalation group (median, not reached vs. 50.7 months, respectively, *P*=0.039) was incorrectly reported.

Correction:

Patients in the escalation group also had longer post-relapse overall survival compared with the non-escalation group (median, not reached vs. 50.7 months, respectively, *P*=0.039).

The escalation group exhibited longer PFS2 (median, 50.4 months vs. 23.5 months, *P* <0.001) and post-relapse overall survival time (median, not reached vs. 50.7 months, respectively, *P*=0.039) than the non-escalation group (Figure 6A–B).

These corrections do not significantly impact the overall findings and conclusions of the paper. We would like to assure readers that the corrected values do not alter the interpretations or validity of the research.
