# Supplementary material for: Sequential treatment escalation improves survival in patients with Waldenstrom macroglobulinemia
Source: Blood Sci. 2024 Jan 17;6(1):e00179. doi: 10.1097/BS9.0000000000000179 (PMC10796142; doi:10.1097/BS9.0000000000000179)
Supplement: Supplementary file 1 [file bs9-6-e00179-s001.pdf]

Supplementary Material

Table S1: The regimens used in this cohort

Regimen	Doses
RCHOP	Rituximab 375 mg/m^2 IV on day 1, cyclophosphamide 750 mg/m^2 IV on day 2, doxorubicin 50 mg/m^2IV on day 2, vincristine 1.4 mg/m^2 IV on day 2 (maximum 2 mg), along with prednisone administered orally at 100 mg a day on days 2-6. These cycles were repeated every 3 weeks.
RCVP	Rituximab 375 mg/m^2 IV on day 1, cyclophosphamide 750 mg/m^2 IV on day 2, vincristine 1.4 mg/m^2 IV on day 2 (maximum 2 mg), along with prednisone administered orally at 100 mg a day on days 2-6. These cycles were repeated every 3 weeks.
DRC	Cyclophosphamide 1000 mg/m^2 IV plus dexamethasone 20 mg IV plus rituximab 375 mg/m^2 IV on day 1 Rituximab was not administered for cycles 1 and 2 of induction therapy in patients with serum IgM level >40 g/L to minimize the risk of an IgM flare. OR rituximab 375mg/m^2 on IV day 8, or PO dexamethasone 20 mg on days 1, 2, 8, 9, 15, 16, 22, 23 and IV or PO cyclophoshamide 500mg/m^2 on days 1, 8, 15. These cycles were repeated every 3 weeks.
BRD	Bortezomib 1.6 mg/ m^2 SC or subcutaneously plus dexamethasone 20 mg IV or orally on days1, 8, 15 plus rituximab 375 mg/m^2 IV on day 11 or 22, respectively. Or the first induction cycle consisted of bortezomib 1.3 mg/m^2 SC on days 1, 4, 8, and 11 followed by 4 cycles of bortezomib 1.6 mg/m^2 SC once per week for 4 weeks with rituximab and dexamethasone in cycles 2 and 5.
RFC	Rituximab 375 mg/m^2 IV on day1, fludarabine 25mg/m^2 IV on day2-4, cyclophosphamide 250 mg/m^2 IV on day2-4. These cycles were repeated every 4 weeks.

BR	Rituximab 375 mg/m^2 IV on day1, bendamustine 90mg/m^2 IV on day2-3. These cycles were repeated every 4 weeks.
BCD	Bortezomib 1.6 mg/m^2 SC on days 1, 8 of the 28-day cycle by subcutaneous or IV or PO dexamethasone 20 mg on days 1, 2, 8, 9, 15, 16, 22, 23 and IV or PO cyclophoshamide 500 mg/m^2 on days 1, 8, 15
Chlorambucil	6-8 mg/d PO on day1-10 and 2 mg/d for maintenance. These cycles were repeated every 4 weeks.
TCD	Thalidomide 100 mg PO QD; cyclophosphamide 300mg/m^2 PO/IV, on day 1,8,15; dexamethasone 20mg PO/IV on day 1,2,8,9. These cycles were repeated every 3 weeks.

Table S2: The most common symptoms at diagnosis

Variable-no. of patients (%)	Patients (N=377)
Fatigue	203 (53.8)
Bleeding	36 (9.5)
Epistaxis	14 (3.7)
Gingival bleeding	9 (2.4)
Dermotorrhagia	13 (3.4)
Edema	16 (4.2)
Hyperviscosity	21 (5.6)
IgM related neuropathy	15 (4.0)
Organ enlargement	13 (3.4)
Hematuria or proteinuria	8 (2.1)
Elevated globulin	26 (6.9)
Other causes	39 (10.3)

Table S3: The shift from first-line to second-line therapy

	**Regimens**	**Number**
Escalation group	R→BTKi	29
	V→BTKi	7
	Cytotoxic drugs→R	9
	Cytotoxic drugs→V	2

	Cytotoxic drugs→BTKi	6
Non-escalation group	R→R	4
	R→V	2
	R→Cytotoxic drugs	6
	V→R	4
	V→V	4
	BTKi→R	2
	BTKi→BTKi	1
	Cytotoxic drugs→Cytotoxic drugs	13

Table S4: The regimens and outcomes in non-escalation group

Regimen	The first-line therapy			The second-line therapy			
	Regimen	Efficacy	PFS1	Regimen	Efficacy	Progressiono or not	PFS2
R→R	RCD	MR	12.5	RCD	VGPR	Yes	12.3
	RCHOP	PR	48.4	RCHOP	-	Yes	23.3
	RCD	-	47.5	RCVP	-	NO	9.1
	RCHOP	PR	69.7	RCHOP	-	NO	1.27
R→V	RCHOP	MR	4.4	VDPACE	SD	Yes	4.9
	RCD	SD	3.6	BCD	MR	Yes	44.4
R→Cytotoxic drugs	RCVP	PR		TCD	MR	Yes	42.6
	RCD	MR		Chlorambucil	PR	Yes	24.3
	RCD	PR		Chlorambucil	PR	No	1.0
	BR	PR		TCD	MR	Yes	10.6

	RCHOP	PR		Fludarabine	-	No	23.1
	RCHOP	-		TCD	SD	Yes	11.1
V→R	BCD	CR	24.1	RCP	-	Yes	39.1
	BCD	SD	14.0	RCD	PR	Yes	15.0
	BCD	PR	34.9	RCD	-	NO	20.9
	BCD	PR	35.3	RCHOP	PR	NO	11.7
V→V	BCD	PR	43.4	BCD	-	Yes	59.3
	BCD	PR	37.8	Bortezomib	-	Yes	34.2
	BD	MR	11.6	BCD	SD	Yes	8.1
	BCD	PR	24.3	BCD	-	Yes	25.0
BTKi→R	Ibrutinib	SD	6.9	IR	PR	No	21.6
	ZID	PR	17.4	BR	-	No	4.9
BTKi→BTKi	Ibrutinib	PR	29.9	Ibrutinib	PR	No	1.67
Cytotoxic drugs→Cytotoxic drugs	M2+Chlorambucil	SD	6.8	CHOP+M	-	Yes	29.7
	EVAD	PR	146.3	FC	SD	No	1.13
	CHOP	PR	60.87	VAD	-	Yes	48.8
	Chlorambucil	-	16.8	TP	-	No	1.7
	CVP	VGPR	26.8	HyperCVAD	-	Yes	40.9
	TCD	PR	22.8	Melphalan	SD	Yes	12.6
	MPT	PR	16.8	Chlorambucil+thalidomide	-	Yes	20.9
	Fludarabine	SD	5.3	CHOP	-	No	5.1
	Chlorambucil	SD	92.3	Fludarabine	PR	No	56.8
	MPT	PR	29.8	CHOP	-	Yes	50.7
	Chlorambucil	MR	36.4	TCD	PR	Yes	37.0

	Melphalan	-	87.8	Chlorambu cil	-	No	79.0
	CHOP	-	72.0	Fludarabine	-	Yes	48.7

RCD: rituximab, cyclophosphamide and dexamethasone; RCHOP: rituximab, cyclophosphamide, doxorubicin, vincristine, and prednisone; RCVP: rituximab, cyclophosphamide, vincristine, and prednisone; BR: rituxmab and bendamustine; VDPACE: Bortezomib, dexamethasone, cisplatin, doxorubicin, cyclophosphamide, and etoposide; BCD: Bortezomib, cyclophosphamide, and dexamethasone; TCD: thalidomide, cyclophosphamide, and dexamethasone; ZID□zanubrutinib, ixazomib, and dexamethasone; IR: ixazomib and rituximab; M2: vincristine and melphalan; FC: fludarabine and cyclophosphamide; EVAD: etoposide, doxorubicin, vincristine, and prednisone; MPT: melphalan, thalidomide and prednisone; TP: thalidomide and prednisone; CR: complete remission; VGPR: very good partial response; MR: minor response; SD: stable disease.

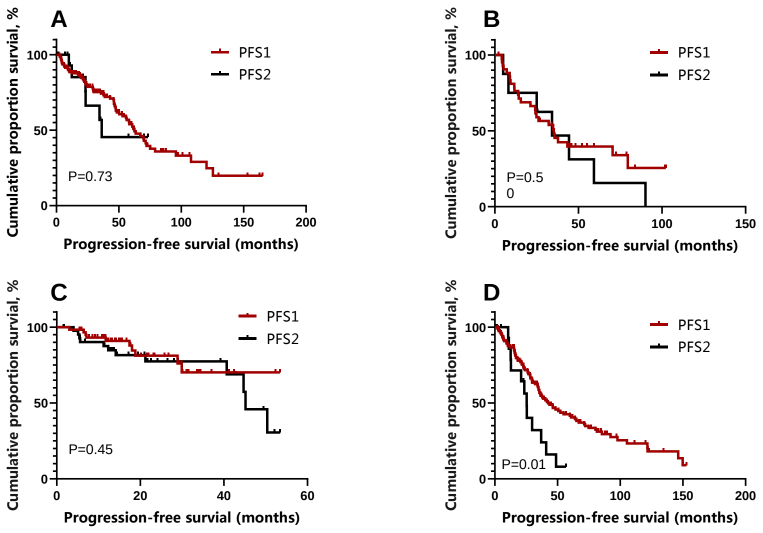

Supplementary Figure 1. The progression-free survival (PFS) 1 and PFS2 of the patients under different treatment regimens. (A) The patients in the R-based group, (B) The patients in theV-based group, (C) The patients in the BTKi-based group, (D) The patients in the cytotoxic drugs group. R, rituximab; V, bortezomib; BTKi, Bruton's tyrosine kinase inhibitors.

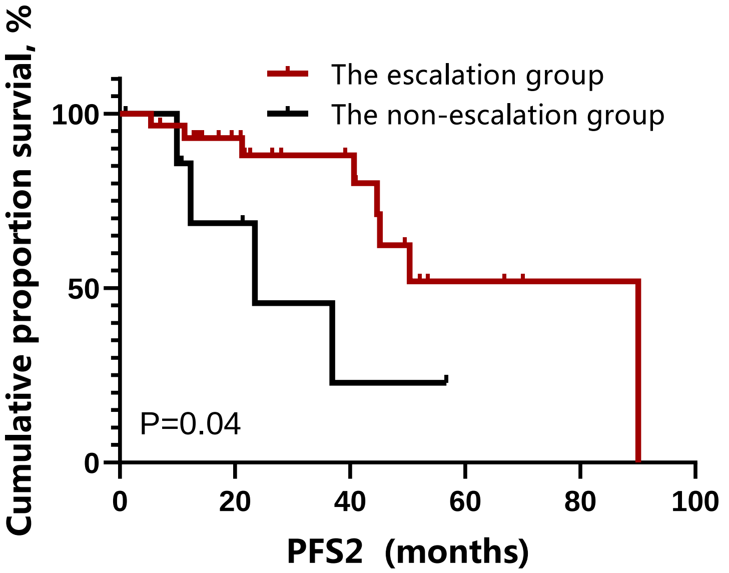

Supplementary Figure 2. The progression-free survival (PFS) 2 of the patients obtaining the major response rate for second-line therapy in the escalation and non-escalation groups.
